# Researching the CNN Collaborative Inference Mechanism for Heterogeneous Edge Devices

**DOI:** 10.3390/s24134176

**Published:** 2024-06-27

**Authors:** Jian Wang, Chong Chen, Shiwei Li, Chaoyong Wang, Xianzhi Cao, Liusong Yang

**Affiliations:** College of Computer and Control Engineering, Northeast Forestry University, Harbin 150040, China; wangj.icec@nefu.edu.cn (J.W.); cc235650@163.com (C.C.); 15666016740@163.com (S.L.); wangchaoyong666@163.com (C.W.); cxz18525357537@163.com (X.C.)

**Keywords:** edge, heterogeneity, model partitioning, CNN inference, pipeline parallelism

## Abstract

Convolutional Neural Networks (CNNs) have been widely applied in various edge computing devices based on intelligent sensors. However, due to the high computational demands of CNN tasks, the limited computing resources of edge intelligent terminal devices, and significant architectural differences among these devices, it is challenging for edge devices to independently execute inference tasks locally. Collaborative inference among edge terminal devices can effectively utilize idle computing and storage resources and optimize latency characteristics, thus significantly addressing the challenges posed by the computational intensity of CNNs. This paper targets efficient collaborative execution of CNN inference tasks among heterogeneous and resource-constrained edge terminal devices. We propose a pre-partitioning deployment method for CNNs based on critical operator layers, and optimize the system bottleneck latency during pipeline parallelism using data compression, queuing, and “micro-shifting” techniques. Experimental results demonstrate that our method achieves significant acceleration in CNN inference within heterogeneous environments, improving performance by 71.6% compared to existing popular frameworks.

## 1. Introduction

With the rapid development of cloud and edge computing, as well as the widespread application of artificial intelligence, CNNs have been extensively utilized in intelligent applications. In traditional cloud computing, most data are processed in the cloud [1], and the resulting high latency, high bandwidth, and high computational-resource demands urgently need to be addressed. Additionally, as artificial intelligence rapidly evolves, the complexity of CNN inference tasks has exploded, and reliance on central clouds for CNN inference faces unstable transmission links, excessive communication costs [2], and data security and privacy issues [3]. The explosive growth in technologies such as 5G and big data has spurred massive demand for smart edge terminal devices, driving industry innovation and implementing intelligent edge architectures. To address the issues above, edge computing paradigms like mobile edge computing and fog computing have emerged. These paradigms can reduce network transmission latency, meet the intensive computing needs at the network edge [4], and handle the complexity of CNN inference tasks locally. By processing data locally at the source, edge computing helps enable real-time prediction, enhanced scalability, and improved latency and privacy [5]. Due to the advantages of edge computing in terms of latency, bandwidth, and security, it provides more effective technical support for many applications that require real-time security [6].

Edge terminal devices are typically limited in computing power and storage, and the neural network inference process is computation-intensive. Executing complex deep learning models on a single resource-constrained edge terminal device can lead to longer inference times. To overcome the limited computational resources of edge terminal devices, neural-network acceleration techniques such as network pruning [7], parameter quantization [8], and weight sharing [9] can increase neural-network computation speed. However, these techniques are complex in practical applications, and excessively compressing structurally complex CNN models to speed up inference could significantly degrade model inference accuracy, while offloading models to edge servers can introduce latency, bandwidth, and privacy issues.

Edge-to-edge collaboration, aggregating the computing resources of multiple edge terminal devices to collaboratively execute CNN inference tasks, can effectively address the aforementioned issues. However, the collaborative execution of inference tasks among edge terminal devices still face several challenges. First, the complexity of CNN models, with their numerous layers and parameters, requires substantial computational resources and storage space for inference tasks. Deploying and executing these complex models on edge devices can lead to performance issues. Second, the heterogeneity of edge devices results in varying computational capabilities, storage capacities, and network bandwidths. This means that the same task may have significantly different performance outcomes on other devices, necessitating careful consideration of these differences to achieve optimal performance and resource utilization. Furthermore, the dynamic nature of IoT systems means that the state of devices and networks in edge computing environments may constantly change, impacting task execution and the accuracy of results. In the context of edge computing, there is an urgent need to fully utilize edge terminal devices for inference. This will enable edge detection-based applications and reduce over-reliance on the cloud. This paper proposes a distributed deep-learning collaborative inference scheme to complete CNN tasks with guaranteed accuracy by collaborating with edge heterogeneous terminal devices.

The main contributions of this paper can be summarized as follows:

While current model partitioning primarily focuses on device clusters within homogeneous architectures, this paper simulates the heterogeneity of IoT devices in edge environments and proposes a CNN collaborative inference framework among heterogeneous device clusters. Based on the heterogeneous edge terminal devices, the pre-trained model architecture is partitioned, and each edge terminal-device node loads the original sub-model architecture and weight information to prevent loss of CNN inference accuracy.

A queuing mechanism and dual compression are introduced to construct a collaborative pipeline job among heterogeneous devices, reducing communication overhead between devices, enhancing the utilization of edge computing resources, and increasing the throughput of collaborative inference on heterogeneous edge devices.

The methods proposed in this paper are complementary to existing deep learning frameworks and model compression techniques and can be integrated with other deep learning frameworks and model compression techniques to further accelerate CNN inference.

## 2. Related Work

The literature [10] proposes an IoT-based intelligent solution for household cooking-oil collection, highlighting the advantages of edge computing over cloud computing. Unlike traditional cloud computing paradigms, this solution relies on edge node infrastructure for data processing. Implementing CNN models on edge terminal devices involves layer partitioning and deployment near the user to ensure fast execution. This is similar to our proposed pre-partitioning deployment method based on key operational layers; however, our Hecofer method further optimizes the data processing and transmission processes, thereby reducing overall system latency. The literature [11] designed a local distributed computing system that partitions a trained DNN (Deep Neural Network) model across multiple mobile devices to accelerate inference. By reducing the computational cost and memory usage of individual devices, it was the first to implement collaborative DNN inference tasks across multiple IoT devices. This method has limitations in handling non-chain-structured models in complex environments. The literature [12] proposed a deep reinforcement learning-based distributed algorithm to optimize computational offloading with minimal latency. The literature [13] proposes an optimization algorithm for offloading decisions and computational resource allocation. The algorithm is based on dual Q-Learning and aims to reduce the maximum delay consumption between individual devices. The literature [14] proposes a multi-user computation offloading and resource-allocation optimization model to minimize the overall system latency. Although these methods have made progress in optimizing latency, they seldom take into account the heterogeneity of devices. The literature [15] presents a distributed dynamic task offloading algorithm based on deep reinforcement learning to optimize the current workload of edge clients and edge nodes. The literature [16] proposes using segment-based spatial partitioning to divide inference tasks, using a layer-fusion parallelization method to empirically divide the CNN model into four fused blocks, each with approximately the same number of layers. However, these methods exhibit limitations when handling complex non-chain-structured models. The literature [17], based on the availability of computational resources and current network conditions, uses a fused convolutional-layer approach with spatial partitioning technology to select the optimal degree of parallelism. The literature [18] used layer fusion, combined with inference tasks, to dynamically adjust and achieve load balancing on heterogeneous devices, balancing computational latency and communication latency. Our Hecofer method more effectively achieves a balance between computation and communication through the combined use of data compression and “micro-shifting” techniques, further optimizing system performance. The literature [19] employed a novel progressive model-partitioning algorithm to handle complex layer dependencies in non-chain structures, partitioning model layers into independent execution units to create nearly new model partitions. Reference [20] proposes a method for neural-network collaborative computing using partitioned multi-layer edge networks. The study establishes a time-delay optimization model to identify the optimal partitioning scheme. These methods have achieved some success in handling non-chain-structured models, but they present certain complexities in practical deployment. The literature [21] adaptively implements vertically distributed inference based on CNNs in a resource-constrained edge cluster. The literature [22] proposes CoopAI, which distributes DNN inference to multiple edge end devices and allows them to preload the necessary data to perform parallel computational inference without data exchange. The literature [5] proposed an inter-device collaborative edge-computing framework, using weight pruning to deploy models to edge terminal devices. The literature [23] introduced a distributed framework for edge systems, Edge Pipe, using pipeline parallelism to accelerate inference, facilitating the operation of larger models. Given the limitations in computational resources and storage of edge terminal devices, existing mechanisms usually assume a chained model structure; however, modern deep-learning models are more complex, often involving non-chain structures. Reference [24] introduces AutoDiCE, a system designed to partition CNN models into a set of sub-models that are collaboratively deployed across multiple edge devices. This approach enhances overall system throughput by optimizing model partitioning and deployment strategies. AutoDiCE offers improvements for both chain-structured and non-chain-structured models, but it does not fully consider the variability in the computational capabilities of devices in edge environments. The literature [25] explores pruning models and task partitioning between edge terminal devices and servers to better adapt to system environments and edge server capabilities, maximizing collaborative inference across edge devices. However, model pruning methods will inevitably reduce the accuracy of model inference. The literature [26] designs a feature compression module based on channel attention in CNN, which selects the most important features to compress intermediate data, thereby accelerating device-edge collaborative CNN inference.

## 3. System Model and Problem Formulation

The definitions of important symbols in this usage section are summarized in Table 1.

### 3.1. System Model

**Figure 1:**
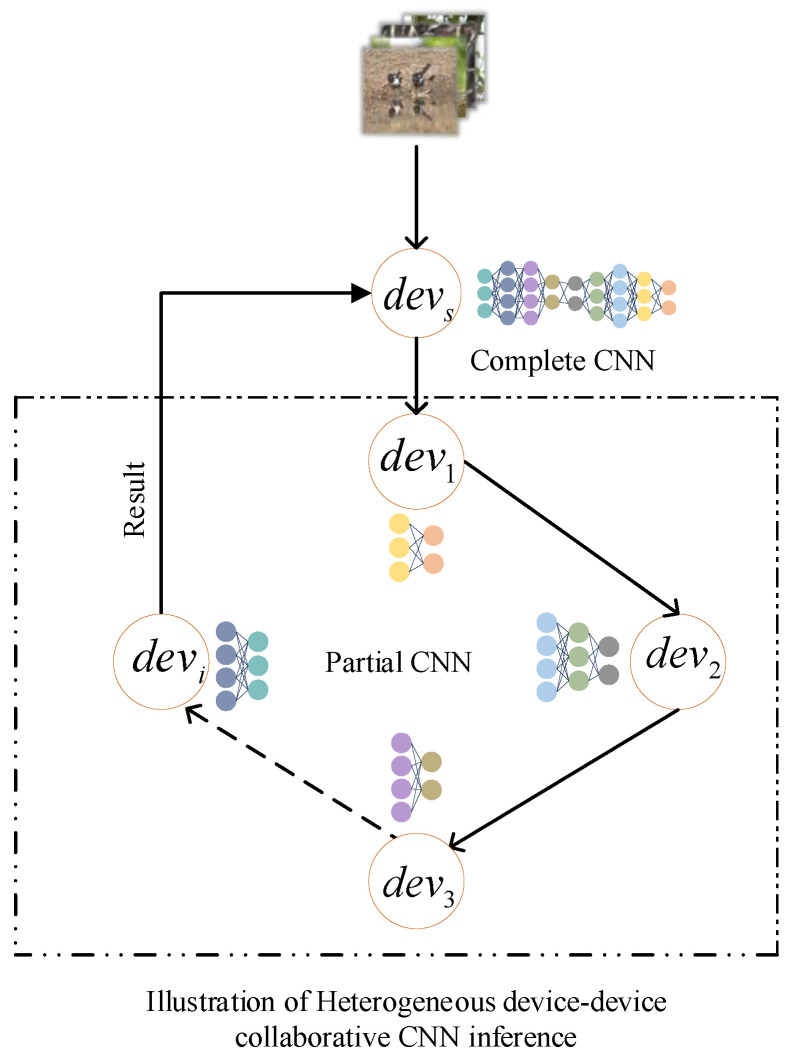
Device-device collaborative CNN-inference execution framework.

To address the challenges faced in edge computing scenarios, where computational and storage resources are limited, making it difficult for a single edge terminal device to efficiently execute computationally intensive inference tasks, we propose a collaborative inference method tailored for heterogeneous edge terminal devices. The overall architecture is shown in Figure 1, which illustrates the edge-to-edge collaborative inference scheme.

In Figure 1, $dev_{i}$ is the edge node device and $dev_{s}$ is the main node device. The master node device $dev_{s}$ replaces the traditional cloud server, reducing the distance between devices and the server. For a CNN inference task m, such as object detection, m is composed of L layers, each of which can be considered a sub-model. Given the number of available edge terminal devices N, the CNN is partitioned by the $dev_{s}$ into R sub-models (R ≤ L), and each sub-model r is assigned to an edge terminal device $dev_{i}$ for execution. During micro-batch inference, the edge main node $dev_{s}$ receives data from the user end and transmits it to the next target node $dev_{i}$. Since the selected device is responsible only for a portion of the original model’s inference, each edge terminal device node $dev_{i}$ must transmit the intermediate output data to the next target device node $dev_{i+1}$, until the CNN inference task is completed. The final-stage node device then transfers the result back to the main node device $dev_{s}$.

### 3.2. Time Prediction Model

**Figure 2:**
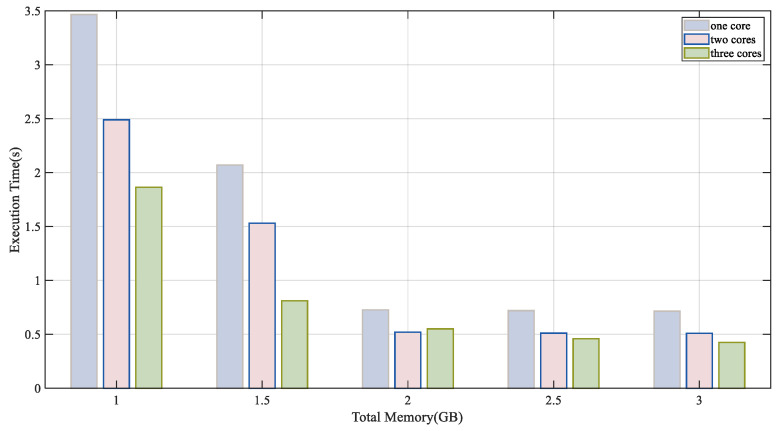
ResNet50 inference latency under different resource models.

As illustrated in Figure 2, we tested the inference latency of ResNet50 under 15 different resource conditions (number of cores, memory). The latency of executing inference tasks significantly decreases as device resources increase, indicating that device capability disparities significantly affect the inference latency of CNN tasks. Our experiments suggest that CNN partitioning should consider the heterogeneous capabilities between devices to fully utilize the computational resources of edge terminal devices to minimize inference latency.

As the device performs CNN inference, the computation is primarily concentrated in the model’s convolutional and fully connected layers. And the computation time for convolutional and fully connected layers correlates with the number of floating-point operations per second (FLOPs). By calculating FLOPs, we can estimate the computation time for the model’s convolutional and fully connected layers, providing a basis for the initial partitioning of the CNN model. The literature [27] provides the formulas for calculating FLOPs for convolutional and fully connected layers, as shown in Formulas (1) and (2), where H and W represent the height and width of the feature map; $C_{in}$ and $C_{out}$ represent the number of input and output channels for the convolution; K represents the size of the convolutional kernel; and I and O represent the input and output dimensions for the fully connected layer.

(1) $$F_{\mathrm{FLOPs}}=2HW(C_{in}K^{2}+1)C_{out}$$

(2) $$F_{\mathrm{FLOPs}}=(2I-1)O$$

The estimated model for calculating time based on FLOPs is shown in Formulas (3)–(5), where x represents FLOPs; $k_{devi}$ represents the computational capability of the device; y represents the computational time of the device; and b is the inherent time overhead. Multiple computations of convolution and fully connected operations are performed and averaged across edge device nodes, utilizing varied input-feature map dimensions (H×W), input and output channel numbers ($C_{in},\;C_{out}$), and input and output sizes (I, O). Multiple sets of FLOPs and computation times are recorded, and estimated models are obtained using the least squares method to gauge the relative computational power of heterogeneous devices.

(3) $$y_{devi}=k_{devi}x+b$$

(4) $$k_{devi}=\frac{n\sum_{i=1}^{n}x_{i}y_{i}-\sum_{i=1}^{n}x_{i}\sum_{i=1}^{n}y_{i}}{n\sum_{i=1}^{n}x_{i}^{2}-(\sum_{i=1}^{n}x_{i}{)}^{2}}$$

(5) $$b={\bar{y}}_{devi}-k_{devi}\bar{x}$$

**Figure 3:**
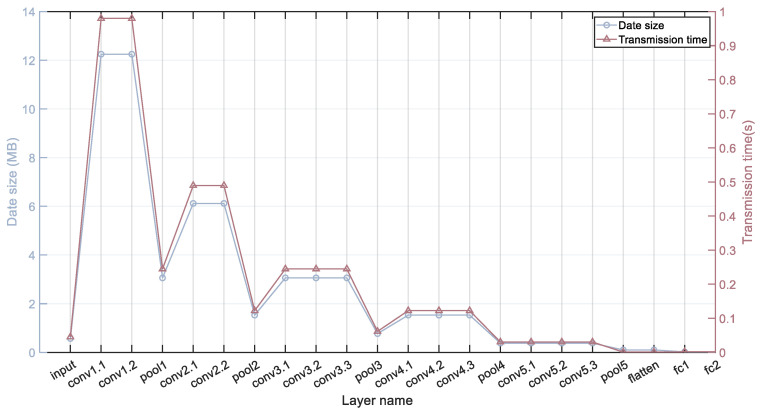
VGG16 output data size and transmission delay of each layer.

In device-to-device collaborative inference, the transmission overhead of intermediate data remains a non-negligible factor. As shown in Figure 3, we simulate the edge terminal-device computing environment in an existing low-bandwidth experimental environment, using VGG16 as an example, to statistically analyze the size and transmission delay of model layer outputs. In Figure 3, the transmission delay of model layer outputs shows a high positive correlation with the data volume of layer outputs.

The experimental results indicate that in bandwidth-constrained harsh environments, CNN partitioning should consider the size of model-layer output data to mitigate the impact of transmission latency on system throughput, particularly by avoiding intermediate layers with large output data.

Furthermore, to further reduce the data transmission volume between devices $dev_{i}$ and $dev_{i+1}$ in device collaborative inference, we employ a data compression algorithm to reduce communication demands. Thus, during device-to-device collaborative inference, the total delay $T_{total}^{dev_{i}}$ for a single node device $dev_{i}$ mainly consists of three parts:

The data acquisition latency $T_{get}$;

The computation latency $T_{comp}$ of edge device $dev_{i}$;

The transmission latency $T_{comm}$ to the next edge device $dev_{i+1}.$

Since the time for compression and decompression is usually negligible, it is often disregarded.

(6) $$T_{total}^{dev_{i}}=T_{comp}^{devi}+T_{comm}^{devi}+T_{get}^{devi}$$

where the size of $T_{get}^{devi}$ depends on the overall delay $T_{total}^{dev_{j}}$ of the previous device $dev_{j}$. In addition, in the micro-batch inference process, if the total inference delay of the current device is less than the total inference delay of its target node device, the current device can send intermediate data into the queue of the target device ahead of time after completing the inference task, for the target device to continue to complete the inference task, saving the target device’s wait for acquisition time. Therefore, when Equation (7) is satisfied, Equation (6) can be simplified to Equation (8).

(7) $$T_{total}^{devj}<T_{total}^{devi}\;(0<j<i)$$

(8) $$T_{total}^{devi}=T_{comp}^{devi}+T_{comm}^{devi}$$

This means that minimizing the overall delay on device $dev_{i}$ translates to minimizing the computation and communication delays.

### 3.3. Pipeline Model

The advantage of multi-device collaborative inference is to reduce the computational load on a single device and lower the inference delay of the task. However, when reducing the computational delay $T_{comp}$, the transmission of intermediate data from device $dev_{i}$ to $dev_{i+1}$ introduces an inter-device communication transmission delay $T_{comm}$. Therefore, it is necessary to design a collaborative inference mechanism among multiple edge terminal devices, appropriately partitioning and distributing CNN inference tasks to effectively balance the computational delay $T_{comp}$ and communication delay $T_{comm}$, to minimize the overall delay $T_{total}$ of the CNN inference task.

**Figure 4:**
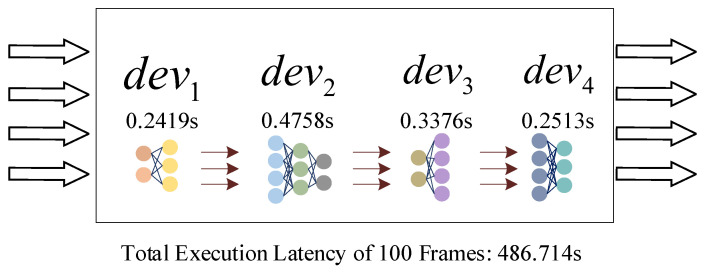
Schematic diagram of pipeline model processing.

After partitioning and deploying the model, each edge device is responsible for completing a portion of the original model’s inference. To achieve efficient collaborative inference at the edge and shorten the inference time of tasks, we introduce pipeline processing. As shown in Figure 4, edge terminal devices can independently perform inference on their partitioned sections.

Specifically, for a sequence of continuous inference tasks c, edge device $dev_{i}$ first completes the first inference task and transmits the intermediate data to $dev_{i+1}$, which then continues to execute the first inference task. At this moment, $dev_{i+1}$ is executing the first inference task. When the second inference task arrives, device $dev_{i}$ immediately begins executing the second inference task. Devices are capable of concurrently processing tasks at different stages, thus reducing idle time. Thus, when multiple continuous inference tasks are input, devices efficiently execute inference tasks in a pipeline parallel manner. Unfortunately, our experiments have verified that the device with the longest execution time becomes the bottleneck in device-to-device collaborative inference.

This paper partitioned the VGG19 model into four parts, each deployed on four different edge heterogeneous devices for collaborative execution of target classification tasks. The time taken by each device to complete one round of inference was, respectively, 0.2419 s, 0.4758 s, 0.3376 s, and 0.2513 s. The total time for 1000 rounds of inference was 486.714 s, approximately 1000 times the execution time of the longest device. Therefore, to fully utilize the computational resources of device-to-device systems and improve system throughput, it is necessary to minimize the bottleneck delay in the system.

## 4. CNN Model-Partitioning Deployment and Optimization Methods

### 4.1. CNN Model-Partitioning Deployment and Optimization Methods

In the context of model partitioning, this paper introduces a pre-partitioning method called Hecofer, specifically designed for CNN inference models and targeting key operator layers. Formulas (1) and (2) provide the computation methods for FLOPs in convolutional and fully connected layers. Layer communication delays are estimated based on the size of each layer’s output data combined with communication bandwidth. The computational capacities of heterogeneous edge terminal devices are quantified using benchmark tests and Formulas (3) and (5). The computational capabilities of heterogeneous edge terminal devices are normalized to homogenize them. Hecofer uses a parameterized performance-prediction model for multiple types of CNN layers and heterogeneous edge terminal devices, identifying key operator layers for model partitioning under heterogeneous computational power.

Traditional model partitioning typically assumes that the model exists in a chain structure, where each layer strictly depends on the output of the previous layer. However, actual model computation graphs may have multiple parallel paths, with one layer possibly depending on several previous layers, which increases the complexity of model partitioning. Hecofer addresses this issue by supporting not only linear structures but also non-linear structures. The model-partitioning approach is primarily based on the topological structure of a directed acyclic graph (DAG). It partitions the DAG structure of the CNN model into key operator nodes, traverses the pre-partition nodes of the initial model, and identifies the starting and ending points for partitioning to construct the partitioned sub-model. The specific division method is shown in Algorithm 1:
**Algorithm 1:** Model Partitioning1. Input:  model: original model  layer_partitions: List of pre-partitioned model layer names 2. Output:  split_models: List of sub-models after partition 3. Initialize an empty model list: models = [] 4.    for p = 0 to (len(layer_partitions) + 1) do 5.     if p == 0 then 6.      Save layer_partitions[p] to start 7.     else 8.      Save layer_partitions[p − 1] to start 9.     end if 10.      if p == len(layer_partitions) then 11.      Set end to model output 12.      Print model.output 13.      else 14.    Set end to layer_partitions[p] 15.      end if 16.    Construct submodel part ← construct_model(model,start,end,part_name) 17.    split_models ← part 18.    return split_models 19.    end for

Algorithm 2 introduces the deployment process for sub-models based on key operator layers. In the initial phase, the edge terminal device $dev_{i}$ acts as a server, autonomously waiting for a connection from the main device $dev_{s}$. Initially, $dev_{s}$ establishes a socket to transmit the model’s weights and structural information to $dev_{i}$. Once the connection is established, edge terminal device $dev_{s}$ creates sockets for model weights and architecture, transmitting sub-model information to $dev_{i}$. Edge terminal device $dev_{i}$ loads model architecture and weight information from $dev_{s}$, instantiating a sub-model with the correct architecture and weights. During the socket information parsing, $dev_{i}$ also acquires the routing information of the next edge terminal device node $dev_{i+1}$ in the inference chain. This allows $dev_{i}$ to forward the intermediate inference results to the next device node $dev_{i+1}$. For the last device node in the inference chain, the routing information of its next node points to the main device $dev_{s}$, ensuring that $dev_{s}$ can correctly receive the final inference results after initiating the inference task. Through this process, the partitioned deployment and inference tasks of the entire CNN model are efficiently executed across multiple edge terminal devices, ensuring scalability and flexibility in model deployment.
**Algorithm 2:** Hecofer Deployment1. Input:  split_models: List of sub-models after segmentation  deviIPs: List of IP addresses of edge heterogeneous devices 2. Output:  None (implementation model deployment) 3. for i = 0 to len(split_models) − 1 do 4.     Set weights_sock to non-blocking mode 5.     Set the weights_sock timeout period 6.     model_json ← split_models[i] 7.     weights_sock.connect ← (deviIPs[i], port) 8.     if i != len(split_models) − 1 then 9.      nextdevi = deviIPs[i + 1] 10.      else 11.       nextdevi = devisIP 12.      end if 13.      Send weights: send_weights(split_models[i].get_weights(),     weights_sock, chunk_size) 14.      Set model_sock to non-blocking mode 15.      Set the model_sock timeout period 16.      model_sock.connect ← (deviIPs[i], port) 17.      Send nextdeviIP to deviIPs[i] 18.      Monitor the model_socket waiting for acknowledgement 19.    end for

### 4.2. Hecofer Optimization Algorithm

Partitioning the CNN model DAG structure based on key operator layers reduces the complexity of searching for the optimal model-partitioning strategy. However, the efficiency of collaborative inference under this method still needs improvement. To enhance the overall performance of collaborative inference among heterogeneous edge terminal devices, this paper designs the Hecofer optimization algorithm.

In the device-to-device collaborative paradigm, edge terminal devices only need to load smaller sub-models, are closer to the data source, and have shorter physical transmission distances, theoretically resulting in smaller communication delays. However, as participating devices need to communicate, this introduces communication time between devices. Formula (8) indicates that under the edge-collaboration paradigm, the execution delay of device $dev_{i}$ is determined by both $T_{comp}^{devi}$ and $T_{comm}^{devi}$. Typically, when multiple devices perform collaborative inference, data compression helps reduce data volume and enhance transmission efficiency due to network and resource limitations. For instance, a data volume of 0.57 MB is compressed to 0.28 MB during transmission in a collaborative inference task involving ResNet50 across three heterogeneous edge devices, achieving a compression rate of approximately 49%.

Our experiments confirm that the device with the longest execution time becomes the bottleneck in device-to-device collaborative inference. To balance $T_{total}^{devi}$ across heterogeneous devices, this paper proposes a “micro-shifting” algorithm based on extremities. After partitioning based on key operator layers, a “micro-shift” adjustment is made for the edge terminal-device node that takes the longest in pipeline operations, reducing the “short-board effect” during pipeline parallelism. The edge main device $dev_{s}$, in each operation, performs a forward layer-offloading task for the device node with the longest execution time, thereby shortening its execution delay. The offloaded layer is moved to an adjacent device node with a shorter execution time, minimizing execution time discrepancies between devices.

Ideally, the execution delays of all edge terminal-device nodes would be identical. Even though “micro-shifting” adjustments can somewhat reduce the bottlenecks in pipeline parallelism, the $T_{total}^{devi}$ of devices remains unequal. To address the idle time caused by devices waiting for output data from preceding devices, the Hecofer optimization method introduces a queue mechanism. As an example, the VGG19 model is divided into four parts and deployed across four different edge devices. The time required for each device to complete one inference cycle is 0.2419 s, 0.4758 s, 0.3376 s, and 0.2513 s, respectively. In this scenario, the edge master device performs layer offloading from the second device to reduce its execution delay. The offloaded layers are transferred to the first device, balancing the execution time.

The queue mechanism allows devices to preemptively receive and store subsequent inference requests while executing the current task, facilitating continuous processing, reducing wait times, and enhancing the overall system throughput. Specifically, each edge device is integrated with a queue for storing multiple inference requests. Once a device completes the current task, it transmits the intermediate results to the next target device and retrieves the next task from the queue for processing. This mechanism prevents devices from waiting for the current task to be completed before receiving new tasks, thereby achieving more efficient inference processing.

## 5. Numerical Results

This section demonstrates the performance of the proposed algorithm. It first describes the experimental setup of this study. Then, the evaluation results are analyzed from different perspectives. Finally, the proposed Hecofer method is compared with the local benchmark inference and existing popular methods, evaluating the performance in terms of latency, throughput, and speedup ratio.

### 5.1. Experimental Environment

Hecofer is implemented using the deep-learning framework TensorFlow. The experiments were conducted using two PCs, one with an Intel(R) Core(TM) i7-12700H (Intel Corporation, Santa Clara, CA, USA) 2.3 GHz with 16 GB RAM and the other with an Intel(R) Core(TM) i7-9700 CPU (Intel Corporation, Santa Clara, CA, USA) H 3.0 GHz with 12 GB RAM. We virtualized 4 Ubuntu 18.04 and 4 Ubuntu 22.04 systems, simulating a collaborative computing environment among eight heterogeneous device terminals under a 100 Mbps bandwidth by assigning different numbers of processor cores and RAM to each virtual machine.

### 5.2. Experimental Dataset and Network Model

Table 2 provides details of several common CNN network models, including the number of parameters, model size, and GFLOPs data for each model. The network models used in this study are the chain-structured VGG16 and VGG19, as well as the non-chain-structured ResNet50.

### 5.3. Evaluation Metrics

This section outlines key evaluation metrics for assessing the performance of the Hecofer method, including total inference latency, inference throughput, and inference speedup:

Total Inference Latency: this encompasses the total time from initiating CNN inference to transmitting the results from the edge terminal device to the main edge device.

Inference Throughput: The system defines a fixed time window during which it records the number of inference cycles completed. This count is then converted into the number of inferences per unit time.

Inference Speedup Ratio: S is defined as the ratio between the total inference time $T_{local}$ for local inference and $T_{Hecofer}$ for the method proposed in this paper.

(9) $$S=\frac{T_{Hecofer}-T_{local}}{T_{local}}\times 100\%$$

## 6. Experimental Results and Analysis

**Figure 5:**
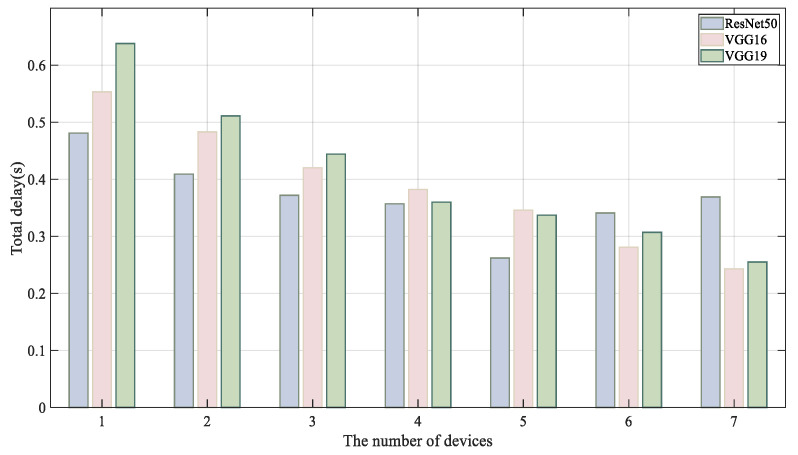
Total inference delay under different number of devices.

Figure 5 illustrates the performance evaluation of the Hecofer method using varying numbers of edge devices. The scenario with a single device represents the baseline inference latency, while systems involving two to seven devices demonstrate the total inference latency during collaborative inference across multiple heterogeneous devices.

For VGG16 and VGG19 models, collaborative inference with seven devices achieves optimal performance. However, the benefits of multi-device collaboration in inference are limited. Taking ResNet50 as an example, when the number of devices increases to six, the overall time cost starts to exceed the benefits of pipeline parallelism. This is because increasing the number of collaborating devices can reduce the computational latency per device but also introduce significant network overhead. In ResNet50, particularly with sic devices involved, the cost of transmitting intermediate data outweighs the benefits of parallel computation.

**Figure 6:**
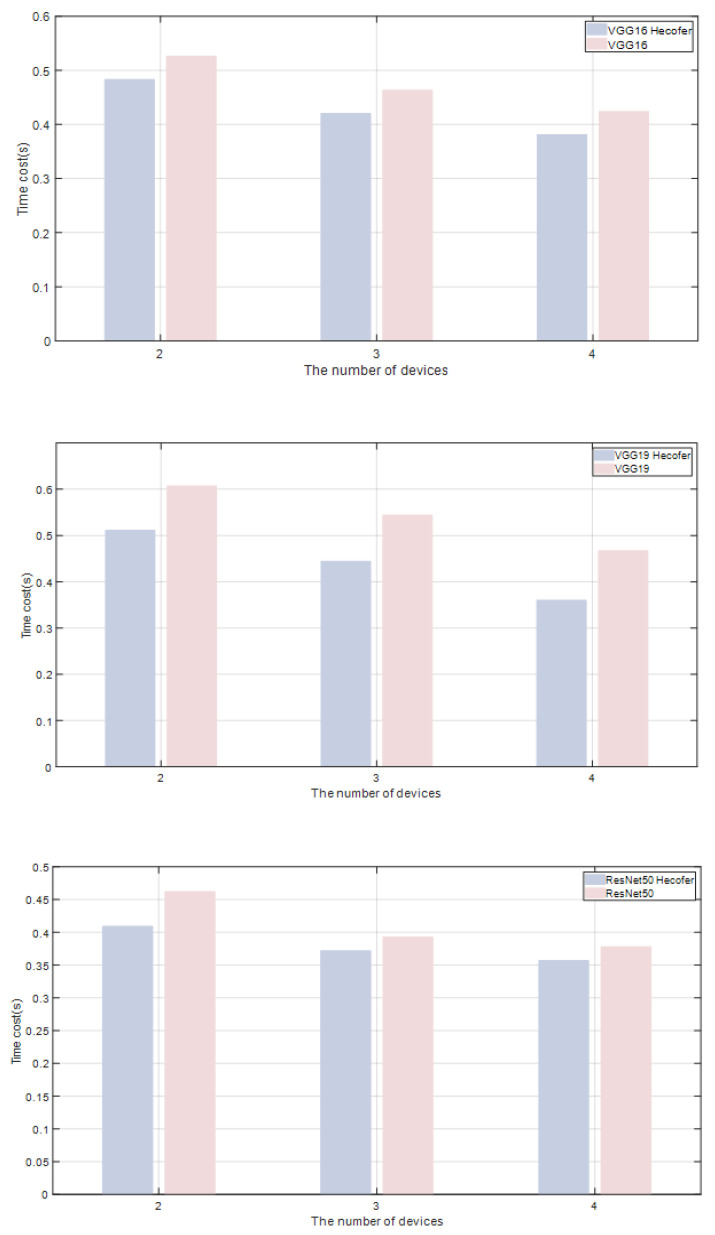
Comparison between Hecofer and equal-layer partitioning.

Figure 6 illustrates a comparison between model-layer partitioning and the Hecofer partitioning method under different levels of device collaboration. The analysis covers the impact of these two partitioning approaches on the overall inference latency when using two, three, and four devices for collaborative inference. Each bar in the figure represents the total inference latency of the respective model across varying numbers of devices. The bars corresponding to the Hecofer method depict lower latency levels, indicating its performance advantage in heterogeneous device environments with identical device configurations.

**Figure 7:**
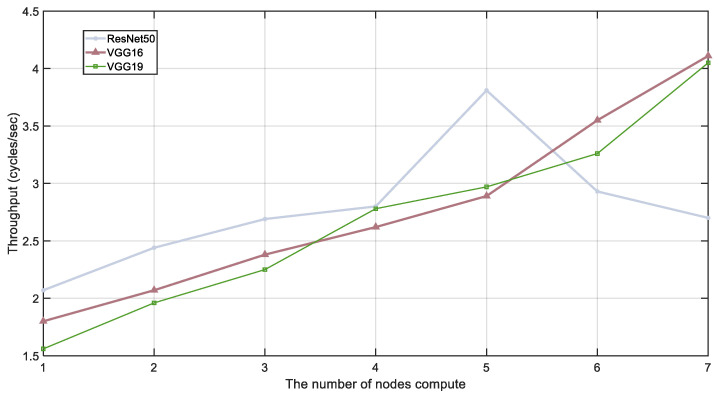
Model throughput under different number of devices.

Figure 7 illustrates the improvement in system model throughput with varying numbers of edge terminal devices participating in collaborative inference. As the number of collaborative devices increases, the throughput of the VGG16, VGG19, and ResNet50 models surpasses the baseline performance. However, Figure 7 also reveals the impact of network overhead on inference throughput.

Specifically, each point in Figure 7 represents the inference throughput with different numbers of devices participating in collaborative inference. It can be observed that when the number of devices increases from one to four, the throughput of all models improves significantly. However, when the number of devices further increases to five and beyond, particularly for the ResNet50 model, the throughput starts to decline. This indicates that while increasing the number of devices initially enhances system performance, the network overhead eventually surpasses the computational benefits, becoming a bottleneck for system performance.

To validate the advancement of the proposed method, a comparison is made with the method from the literature [28], which uses CORE for simulations with near-zero latency in a local environment. Assuming that Hecofer’s communication latency is negligible, we use the bottleneck delay per round as the actual round inference delay for this analysis.

**Figure 8:**
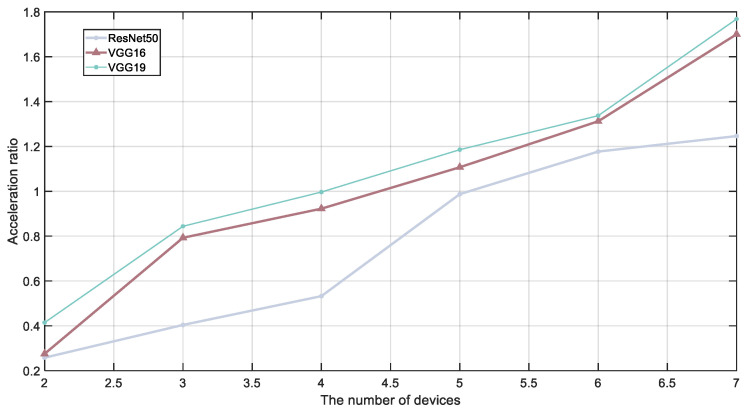
Inference acceleration ratio under different numbers of devices.

Figure 8 simulates the inference speedup ratio under near-zero latency conditions with different numbers of devices. As the number of device nodes increases, the single-round inference latency gradually decreases, and the inference speedup ratio continuously increases. In a four-device setup using a chain-structured model, VGG19’s inference throughput shows a 40% improvement compared to DSE [24]. With seven devices, ResNet50 achieves an inference gain of up to 124.6%, significantly higher than 53% under DEFER [28]. VGG19 and VGG16 reach gains of 170% and 176.8%, respectively. This also highlights Hecofer’s significant advantage for computation-intensive models like VGG19.

These results demonstrate the high versatility and flexibility of the proposed Hecofer model partition-deployment method. It significantly benefits both chain-structured and non-chain-structured models when tailored for collaborative inference across heterogeneous devices.

## 7. Conclusions

This study investigates the partitioned deployment mechanism of CNN models for heterogeneous edge terminal devices and proposes a pre-partitioned deployment method based on critical operation layers called Hecofer, aiming to minimize the overall system latency. Specifically, the Hecofer method identifies and leverages the essential layers of operation within CNN models to pre-partition and deploy the models, significantly reducing inference latency and increasing system throughput across multiple heterogeneous devices. Compared to local benchmark schemes, the Hecofer method significantly reduces the inference latency and increases the throughput for both chain-structured models (e.g., VGG16, VGG19) and non-chain-structured models (e.g., ResNet50). When compared to traditional equal-layer partitioning methods, Hecofer demonstrates significant advantages in multi-heterogeneous device-deployment environments. Additionally, compared to the technique presented in the literature [24,28], our proposed model partitioning-deployment method substantially enhances system throughput.

It is noteworthy that our research differs from methods such as model compression and knowledge distillation. Model compression reduces computational costs by decreasing the number of parameters, while knowledge distillation trains lightweight models to approximate the performance of the original models. In the future, these techniques could potentially be combined with the Hecofer method to further accelerate CNN inference speed on edge terminal devices through multi-device collaboration.

## Figures and Tables

**Table 1:** List of notations.

Notation	Description
m	CNN Inference Task
L	Number of layers in the CNN model
N	Number of heterogeneous devices at the edge
${dev}_{i}$	The *i*-th edge collaborative device
$dev_{s}$	The main edge device
R	Number of CNN sub-models
$T_{comp}$	Computational delay
$T_{comm}$	Transmission delay
$T_{total}$	Total latency of the reasoning task
$T_{l}^{dev_{i}}$	$\mathrm{The}\;comm\left(comp,get,total\right)$ latency of the device $dev_{i}$
r	CNN submodel
c	CNN inference tasks

**Table 2:** Popular DNN models.

Method	Type	Parameters	Model Size (MB)	GFLOPS
AlexNet	CNN	60,965,224	233	0.7
VGG-16	CNN	138,357,544	528	15.5
VGG-19	CNN	143,667,240	548	19.6
ResNet50	CNN	25,610,269	98	3.9
ResNetl01	CNN	44,654,608	170	7.6
ResNetl52	CNN	60,344,387	230	11.3

## Data Availability

Dataset available on request from the authors.
